# Supplementary material for: MetaQTL: a package of new computational methods for the meta-analysis of QTL mapping experiments
Source: BMC Bioinformatics. 2007 Feb 8;8:49. doi: 10.1186/1471-2105-8-49 (PMC1808479; doi:10.1186/1471-2105-8-49)
Supplement: Additional File 7 — MetaQTL Package : jar file and tutorial. This Zip archive contains both the MetaQTL JAR file and the files of the tutorial. [file 1471-2105-8-49-S7.zip › org.inra.metaqtl/doc/index-files/index-22.html]

W-Index


|  |  |  |
| --- | --- | --- |
| **Overview** \| Package \| Class \| Use \| **Tree** \| **Deprecated** \| **Index** \| **Help** | |  |
| **PREV LETTER**   **NEXT LETTER** | **FRAMES**    **NO FRAMES**     **All Classes** |  |


A B C D E F G H I K L M N O P Q R S T U V W X Y Z 

---


## **W**

**wantsValue()** - Method in class org.metaqtl.main.CmdLineParser.Option: Tells whether or not this option wants a value **WARD\_METHOD** - Static variable in class org.metaqtl.algo.HClustAlgorithm: Ward method **WardMethod(double[], double[])** - Static method in class org.metaqtl.algo.HClustAlgorithm: This method performs a hierarchical clustering of the given data points using the Ward's method. **WIDTH** - Static variable in class org.metaqtl.graph.GraphFactory: **WITH\_CHROM\_NAME** - Static variable in class org.metaqtl.graph.MetaGraphPar: **WITH\_COMMON\_MARKER** - Static variable in class org.metaqtl.graph.MetaGraphPar: **WITH\_LEGEND** - Static variable in class org.metaqtl.graph.MetaGraphPar: **WITH\_MAP\_NAME** - Static variable in class org.metaqtl.graph.MetaGraphPar: **WITH\_MARKER\_NAME** - Static variable in class org.metaqtl.graph.MetaGraphPar: **WITH\_MARKER\_POSITION** - Static variable in class org.metaqtl.graph.MetaGraphPar: **WITH\_QTL\_NAME** - Static variable in class org.metaqtl.graph.MetaGraphPar: Add the Qtl name. **write(EMResult, OutputStream)** - Static method in class org.metaqtl.factory.EMResultFactory: **write(EMResult, PrintWriter)** - Static method in class org.metaqtl.factory.EMResultFactory: **write(OutputStream)** - Static method in class org.metaqtl.factory.MetaGraphParFactory: **write(Writer)** - Static method in class org.metaqtl.factory.MetaGraphParFactory: **write(MetaMap, OutputStream)** - Static method in class org.metaqtl.factory.MetaMapSummaryFactory: This methods writes out the summary for a consensus genetic map into a given output stream `stream`. **write(MetaQtlAnalysis, OutputStream)** - Static method in class org.metaqtl.factory.MetaQtlAnalysisFactory: This methods writes out in the given `stream` the results of a QTL meta-analysis. **write(MetaQtlAnalysis, OutputStream)** - Static method in class org.metaqtl.factory.MetaQtlAnalysisModelFactory: **write(MetaQtlAnalysis, Writer)** - Static method in class org.metaqtl.factory.MetaQtlAnalysisModelFactory: **write(MetaQtlAnalysis, OutputStream)** - Static method in class org.metaqtl.factory.MetaQtlAnalysisSummaryFactory: This method writes out the summary for a givene QTL meta-analysis result `analysis` on the given output stream `stream`. **write(MetaQtlModel, OutputStream)** - Static method in class org.metaqtl.factory.MetaQtlModelFactory: **write(MetaQtlModel, Writer)** - Static method in class org.metaqtl.factory.MetaQtlModelFactory: **write(Tree, OutputStream)** - Static method in class org.metaqtl.factory.TreeFactory: This method writes out the given tree `nodes` as an array of `TreeNode` on the given output stream stream. **write(Tree, PrintWriter)** - Static method in class org.metaqtl.factory.TreeFactory: This method writes out the given tree `nodes` as an array of `TreeNode` using the given writer writer. **writeImg(ChromGraph[], File, String)** - Static method in class org.metaqtl.main.MetaMain: **writePar(File)** - Static method in class org.metaqtl.main.MetaMain

---


|  |  |  |
| --- | --- | --- |
| **Overview** \| Package \| Class \| Use \| **Tree** \| **Deprecated** \| **Index** \| **Help** | |  |
| **PREV LETTER**   **NEXT LETTER** | **FRAMES**    **NO FRAMES**     **All Classes** |  |


A B C D E F G H I K L M N O P Q R S T U V W X Y Z 

---
